# Supplementary material for: MetaRibo-Seq measures translation in microbiomes
Source: Nat Commun. 2020 Jun 29;11:3268. doi: 10.1038/s41467-020-17081-z (PMC7324362; doi:10.1038/s41467-020-17081-z)
Supplement: Supplementary file 10 — Supplementary Data 7 [file 41467_2020_17081_MOESM10_ESM.zip › File2/Confidence_VeryHigh_Taxonomy/266611_out.krona.html]

Javascript must be enabled to view this page.

members
magnitude
magnitudeUnassigned
count
unassigned
taxon
rank

266611\_out

5

5
superkingdom
2

5
phylum
201174

5
1760
class

85004
order
5

5
31953
family


SRS024075\_contig\_number\_contig-100\_18269.18269SRS051610\_contig\_number\_contig-100\_8707.46800
5
2
genus
1678

28026
species
3

SRS1041038\_contig\_number\_1102SRS104912\_contig\_number\_contig-100\_3621.80989SRS1055043\_contig\_number\_contig-100\_5484.87629
